# Clinical and cognitive risk factors for psychotic symptoms in 22q11.2 deletion syndrome: a transversal and longitudinal approach

**DOI:** 10.1007/s00787-013-0469-8

**Published:** 2013-09-03

**Authors:** Maude Schneider, Marie Schaer, A. Kadir Mutlu, Sarah Menghetti, Bronwyn Glaser, Martin Debbané, Stephan Eliez

**Affiliations:** 1 Office Médico-Pédagogique Research Unit, Department of Psychiatry, University of Geneva School of Medicine, 1 David Dufour, CP 50, 1205 Geneva, Switzerland; 2 Stanford Cognitive and Systems Neuroscience Laboratory, Stanford University, 1070 Arastradero Road, Palo Alto, CA 94304 USA; 3 Adolescence Clinical Psychology Research Unit, Faculty of Psychology, University of Geneva, 40 Pont d’Arve, 1205 Geneva, Switzerland; 4 Department of Genetic Medicine and Development, University of Geneva School of Medicine, Michel Servet 1, 1206 Geneva, Switzerland

**Keywords:** 22q11DS, Schizophrenia, Positive psychotic symptoms, Hallucinations, Negative symptoms, Mixed models

## Abstract

22q11.2 deletion syndrome (22q11DS) is associated with increased risk for schizophrenia. Better identifying risk factors for the emergence of psychotic symptoms in this population is needed to improve clinical assessment and early interventions. Schizophrenia spectrum disorders, hallucinations and delusions were characterized in an original sample of 104 individuals with 22q11DS. Further analysis of positive and negative symptoms was performed in a subsample of 59 individuals. Finally, longitudinal data available in 56 patients were used to explore the developmental trajectories of psychotic symptoms as well as the associations between psychotic symptoms and cognitive functioning. Schizophrenia spectrum disorders and psychotic symptoms were frequent in adolescent and adults with 22q11DS. The severity of hallucinations and non-persecutory delusional ideas discriminated patients at ultra-high risk for conversion to psychosis. Whereas approximately one-third of patients experienced an emergence of psychotic symptoms during a 4-year interval, 20 % displayed transient symptoms. Individuals with psychotic symptoms were characterized by a lower cognitive functioning in the context of the 22q11DS. The present study adds important data on the characteristics and developmental trajectory of psychotic symptoms in this population. This information may ultimately help clinicians dealing with these patients to reduce the duration of untreated psychosis and improve outcome.

## Introduction

22q11.2 deletion syndrome (22q11DS) is associated with a high risk of developing schizophrenia [1], a psychiatric condition that leads to a high degree of disability, family burden and cost for society [2]. Identifying reliable risk factors for the onset of psychotic symptoms represents an important objective for research on 22q11DS. Furthermore, combining investigations into neurobiological, cognitive and clinical aspects of the syndrome will strengthen our ability to accurately predict risk for schizophrenia. In the present paper, we focus exclusively on clinical and cognitive risk factors, as they translate more easily to clinical implications of the syndrome.

Previous studies suggest that attenuated or brief intermittent psychotic manifestations, such as isolated hallucinations and subthreshold delusional ideas, are frequent in children and adolescents with 22q11DS and represent one of the strongest predictors for the onset of a full-blown psychotic disorder [3, 4]. However, no study to date has investigated in detail the phenomenology and developmental trajectory of psychotic symptoms in this population. Furthermore, although negative symptoms are frequent and often severe in 22q11DS youth [5, 6], their role in the pathogenesis of schizophrenia has been less clearly established. In our opinion, a clear description of these manifestations may improve our ability to predict the onset of schizophrenia and reduce the duration of untreated psychosis in these at-risk patients.

When aiming at accurately predicting the onset of psychotic symptoms, a careful assessment of the cognitive status in these patients may also add valuable information. Indeed, research in schizophrenia shows that deficits in some cognitive areas are already present from childhood and remain relatively independent of positive symptomatology over time [e.g. 7, 8]. In particular, low IQ as well as deficits in executive functioning, working memory and attention has been described as early trait cognitive markers for schizophrenia [9–11]. On the other hand, some cognitive abilities may deteriorate just before or during the onset of full-blown psychosis, thereby representing proximal state markers for schizophrenia. According to Frommann [12], working memory, and verbal and visual memory deficits worsen just before the onset of psychosis, whereas executive dysfunction may reflect a more stable aspect of the disease. In 22q11DS, few longitudinal studies have examined cognitive risk factors for psychotic symptoms. They observed that a decline in verbal IQ, low verbal abilities and executive functioning during childhood predicted the severity of psychotic symptoms later in life [13, 14].

The present study combines cross-sectional and longitudinal data acquired within the 22q11DS Swiss longitudinal study since 2002. Firstly, cross-sectional data collected in a large cohort of patients were used to characterize positive psychotic symptoms and schizophrenia spectrum disorders in this population. As already described in previous studies [e.g. 1, 3, 15], we expected to observe a high prevalence of positive psychotic symptoms and schizophrenia spectrum disorders in adolescents and adults with 22q11DS. Secondly, we examined which dimension of positive and negative symptoms best distinguish individuals at different stages of psychosis (non-prodromal, at-risk mental state and psychotic state). Based on previous publications [3, 16], we hypothesized that the severity of hallucinations would best discriminate between these stages. We also explored if the severity of negative symptoms was increased in patients in an at-risk mental state. Thirdly, using longitudinal data available for a subsample of patients, we characterized the different developmental trajectories of positive psychotic symptoms over time. Indeed, our clinical experience suggests that psychotic symptoms do not recede spontaneously in most 22q11DS patients, but a small percentage of them may experience transient psychotic symptoms. To our knowledge, this has never been examined in previous studies and warrants investigation due to the direct implications for clinical practice. Finally, using the longitudinal dataset, we examined the developmental trajectory of cognitive functioning in participants with and without psychotic symptoms to identify putative trait and state markers for the presence of psychotic symptoms. Based on previous longitudinal studies in 22q11DS and schizophrenia [13, 14], we expected that the presence of psychotic symptoms would be associated with low general intellectual functioning (trait marker) and a decrease in memory functioning and verbal reasoning abilities (state markers).

## Method

### Participants

This cohort has been the subject of previous publications and the global research strategy has already been published elsewhere [e.g. 17]. Briefly, all participants were recruited through advertisements in patient association newsletters and through word of mouth. Written informed consent was obtained for all participants and their parents under the protocols approved by the Institutional Review Board of the Department of Psychiatry of the University of Geneva Medical School. The presence of a 22q11.2 microdeletion was confirmed using quantitative fluorescent polymerase chain reaction (QF-PCR).

The results from the present study were obtained by analysing cross-sectional data in an original sample of 104 individuals with 22q11DS. They were further completed by additional analyses performed on a cross-sectional subsample of 59 individuals and a longitudinal subsample of 56 individuals (see Table 1).

**Table 1:** Sample description

Sample name	*N*	Mean age (SD)	Gender ratio (males/females)	Clinical instruments used
Original sample	104	*m* = 14.83 (SD = 7.53)	47/57	DICA-R, SCID-I, K-SADS-PL
Cross-sectional subsample	59 (61 % of the original sample)	*m* = 16.93 (SD = 5.67)	26/33	SIPS
Longitudinal subsample	56 (58 % of the original sample)	T1: *m* = 16.60 (SD = 6.73)	23/33	BPRS
		T2: *m* = 20.46 (SD = 6.84)		

The original sample of 104 participants with 22q11DS of age 6–44 years [*m*_age_ = 14.83, SD = 7.53; 57 (55 %) females] was used to characterize the prevalence of hallucinations and delusional ideas. The sample was subdivided into three age groups to compare the prevalence of these manifestations across their lifespan: children aged 6–11 years (*N* = 45); adolescents aged 12–17 years (*N* = 39); and adults above 18 years (*N* = 20). Seventeen (16.3 %) participants were receiving psychotropic medication at the time of testing: six were on methylphenidate, six on antipsychotics, five on antidepressants, three on antiepileptic drugs and two on anxiolytic medication.

As data collected in this large sample remained relatively general, we performed more detailed analyses in a cross-sectional subsample of participants using the Structured Interview for Prodromal Syndromes (SIPS; see “Materials”). The SIPS was introduced later into the Geneva longitudinal study research protocol and is not appropriate for use with young children. SIPS data were available for a subsample of 59 participants (*m*_age_ = 16.93, SD = 5.67; 33 (56 %) females]. Patients assessed with the SIPS were significantly older compared with patients who were not assessed with the SIPS (*t* = −3.423, *p* = 0.001), but did not differ regarding gender distribution (χ^2^ = 0.070, *df* = 1, *p* = 0.792), mean full-scale IQ (*t* = −0.515, *p* = 0.608) and mean BPRS positive symptom score (*t* = −1.460, *p* = 0.147). The prevalence of patients receiving psychotropic medication did not significantly differ between the two groups (χ^2^ = 0.527, *df* = 1, *p* = 0.468). The difference in age was due to the fact that the SIPS was not administered to younger children.

Subsequent to cross-sectional analyses, we analysed the evolution of psychotic symptoms in our cohort using the longitudinal data collected to date with the Brief Psychiatric Rating Scale (BPRS; see “Materials”). These analyses enabled examining the developmental trajectories of positive psychotic symptoms as well as the association between positive psychotic symptoms and cognitive functioning. Longitudinal assessments were available for 63 participants, of whom we excluded 7 aged below 12 years at T2 evaluation to limit the probability of false negatives (i.e. participants with no apparent psychotic symptoms who may still develop symptoms later in life). The final longitudinal subsample was therefore composed of 56 patients [*m*_age_ at T1 = 16.60, SD = 6.73; 33 (59 %) females], with a mean interval of 3.86 years between the two visits (SD = 0.69 year, range 2.57–6.00 years). Patients for whom longitudinal data were not available did not differ from patients assessed longitudinally regarding age (*t* = −1.276, *p* = 0.205), gender distribution (χ^2^ = 0.657, *df* = 1, *p* = 0.417), full-scale IQ (*t* = −0.924, *p* = 0.358) and mean BPRS positive symptom score (*t* = −0.921, *p* = 0.359). The prevalence of patients receiving psychotropic medication did not significantly differ between the two groups (χ^2^ = 2.398, *df* = 1, *p* = 0.121).

### Materials

#### Clinical evaluation

All participants and their parents were interviewed separately by a trained psychiatrist (SE). Parents of participants below the age of 18 years were interviewed using the computerized Diagnostic Interview for Children and Adolescents-Revised [DICA-R; 18] to identify the presence of DSM-IV psychiatric disorders in their children. All diagnoses were confirmed during a clinical evaluation of the child. Adult participants and their parents were interviewed using the Structured Clinical Interview for DSM-IV Axis I disorders [SCID-I; 19]. The psychotic disorders supplement of the Schedule for Affective Disorders and Schizophrenia for School-Age Children Present and Lifetime Version [K-SADS-PL; 20] was also used with all participants and their parents to complete the clinical evaluation. The K-SADS-PL rates the presence of hallucinations and delusions using a three-point severity scale (1 = absent; 2 = subthreshold; 3 = definite).

During the participants’ clinical evaluation, two supplementary instruments were used. The Structured Interview for Prodromal Syndromes [SIPS; 21, 22] was administered to assess positive, negative, disorganization and general prodromal symptoms. Before administering the SIPS, the interviewer (SE) clinically assessed if the participant had a sufficient level of abstract thinking to understand and answer to the SIPS items. During the interview, each symptom was rated on a 7-point severity scale (0 = absent to 6 = severe). The presence of a psychotic syndrome or an at-risk mental state was based on the Criteria for Prodromal Syndromes (COPS 3.0). The Brief Psychiatric Rating Scale [BPRS; 23] was administered to assess positive psychotic symptoms. According to Nicholson et al. [24], four BPRS symptoms are used as proxy measures for positive psychotic symptoms in the vast majority of studies: hallucinatory behaviour, suspiciousness, unusual thought content and conceptual disorganization. In the present study, we decided to exclude the conceptual disorganization item, as it might be influenced by the cognitive difficulties experienced by individuals with 22q11DS. The presence of positive symptoms was therefore assessed by the following BPRS items: hallucinatory behaviour, suspiciousness and unusual thought content. Presence of positive symptoms was determined by a score ≥3 on at least one of these items.

#### Cognitive functioning

Cognitive functioning was assessed in all participants at T1 and T2 evaluations. Participants younger than 17 years completed the Wechsler Intelligence Scale for Children III [WISC-III; 25] and the Children’s Memory Scale [CMS; 26]. Participants older than 17 completed the Wechsler Adult Intelligence Scale III [WAIS-III; 27] and the Wechsler Memory Scale III [WMS-III; 28]. WISC-III/WAIS-III index scores (full-scale IQ, verbal IQ, performance IQ, verbal comprehension index, perceptual organization index, and processing speed index) were used as global measures of verbal and visuo-spatial reasoning as well as processing speed. CMS/WMS-III indices (auditory immediate, auditory delayed, visual immediate, visual delayed and auditory recognition delayed) were used as measures of memory functioning.

### Statistical analyses

The first analyses were based on cross-sectional data and performed using SPSS version 19. We first compared the prevalence of schizophrenia spectrum disorders (based on the DICA, SCID-I and K-SADS-PL), definite hallucinations and delusions, and subthreshold hallucinations and delusions (based on the K-SADS-PL) across age groups using Chi-square tests. Hallucinations were then characterized according to their modalities of expression (auditory, visual or combined forms); delusions were characterized according to their thematic forms (referential, persecutory or combined forms).

Patients with SIPS data available (*N* = 59) were divided into three groups based on the COPS 3.0. criteria: presence of a psychotic syndrome, presence of an at-risk mental state and non-prodromal individuals. The severity of the SIPS positive and negative symptoms was compared between the three groups using Kruskal–Wallis tests with pairwise comparisons of category (accounting for multiple comparisons). Non-parametric analyses were used because of the abnormal distribution of the data and sample size differences between the groups.

The second analyses were based on longitudinal data available for a subsample of 56 patients. We first examined the prevalence of different clinical trajectories of positive psychotic symptoms over time: absence of symptoms (i.e. score <3 on the three BPRS items assessing positive symptoms at T1 and T2 evaluation), transient symptoms (i.e. score ≥3 on at least one positive BPRS item at T1 and score <3 on positive BPRS items at T2), emerging symptoms (i.e. score <3 on positive BPRS items at T1 and score ≥3 on at least one positive BPRS item at T2) and enduring symptoms (i.e. score ≥3 on at least one positive BPRS item at T1 and T2).

We then examined the developmental trajectories of cognitive functioning in patients with and without positive psychotic symptoms at T2. A score ≥3 on at least one positive BPRS item was used to determine the presence of positive psychotic symptoms. These analyses were performed using mixed model regression analyses, allowing to model the within-subject factor as a nested variable [29]. Such mixed model analyses allow increased statistical power in longitudinal dataset such as ours, with variable time between visits and a large age range at T1 evaluation. Different random intercept models (constant, linear, quadratic and cubic) were proposed, using the nlmefit function in MATLAB R2011b (MathWorks). We used a Bayesian information criterion (BIC)-based model selection method, as the BIC-based methods are one of the most powerful model selection methods for the mixed models [30]. Then, a likelihood ratio test was used to quantify significant between-group differences in the cognitive trajectories over time. At the end of the analysis, two types of differences can be observed between the groups: shape differences (the trajectories of cognitive functioning in the two groups have a different shape) and intercept differences (the trajectories in the two groups have the same shape (i.e. they are parallel), but have different intercept values).

## Results

### Cross-sectional analyses

#### Prevalence of schizophrenia spectrum disorders and positive psychotic symptoms

The prevalence of schizophrenia spectrum disorders and positive psychotic symptoms was examined in the original sample of 104 individuals. Schizophrenia spectrum disorders were diagnosed in 10 % of individuals (see Table 2). Their prevalence was significantly different between age groups (χ^2^ = 19.782, *df* = 2, *p* < 0.001), with a higher rate among adults. The youngest patient diagnosed with a schizophrenia spectrum disorder was aged 15.

**Table 2:** Frequency of schizophrenia spectrum disorders, definite psychotic symptoms, and subthreshold psychotic symptoms across age groups

	Children 6–11 y.o. (*N* = 45)	Adolescents 12–17 y.o. (*N* = 39)	Adults >18 y.o. (*N* = 20)	Total sample (*N* = 104)	*p*
Any schizophrenia spectrum disorder	0 (0 %)	3 (8 %)	7 (35 %)	10 (10 %)	<0.001
Schizophrenia	0 (0 %)	1 (3 %)	2 (10 %)	3 (3 %)	
Schizoaffective disorders	0 (0 %)	0 (0 %)	3 (15 %)	3 (3 %)	
Other psychotic disorders^a^	0 (0 %)	2 (5 %)	2 (10 %)	4 (4 %)	
Any definite psychotic symptom	0 (0 %)	9 (23 %)	7 (35 %)	16 (15 %)	<0.001
Definite hallucinations	0 (0 %)	9 (23 %)	6 (30 %)	15 (14 %)	
Definite delusions	0 (0 %)	4 (10 %)	5 (25 %)	9 (9 %)	
Any subthreshold psychotic symptom	7 (16 %)	12 (31 %)	8 (40 %)	27 (26 %)	0.080
Subthreshold hallucinations	4 (9 %)	6 (15 %)	4 (20 %)	14 (13 %)	
Subthreshold delusions	4 (9 %)	7 (18 %)	7 (35 %)	18 (17 %)	

^a^Schizophreniform disorder, delusional disorder, brief psychotic disorder, and psychotic disorder NOS

The prevalence of individuals experiencing definite hallucinations and/or delusions in the total sample was 20 % (see Table 1). Again, their prevalence differed between age groups (χ^2^ = 15.866, *df* = 2, *p* < 0.001), with higher rates in adults. The frequency of individuals experiencing subthreshold hallucinations and/or delusions in the total sample was 26 %. The prevalence was not significantly different in the three age groups (χ^2^ = 5.055, *df* = 2, *p* = 0.080).

**Fig. 1:**
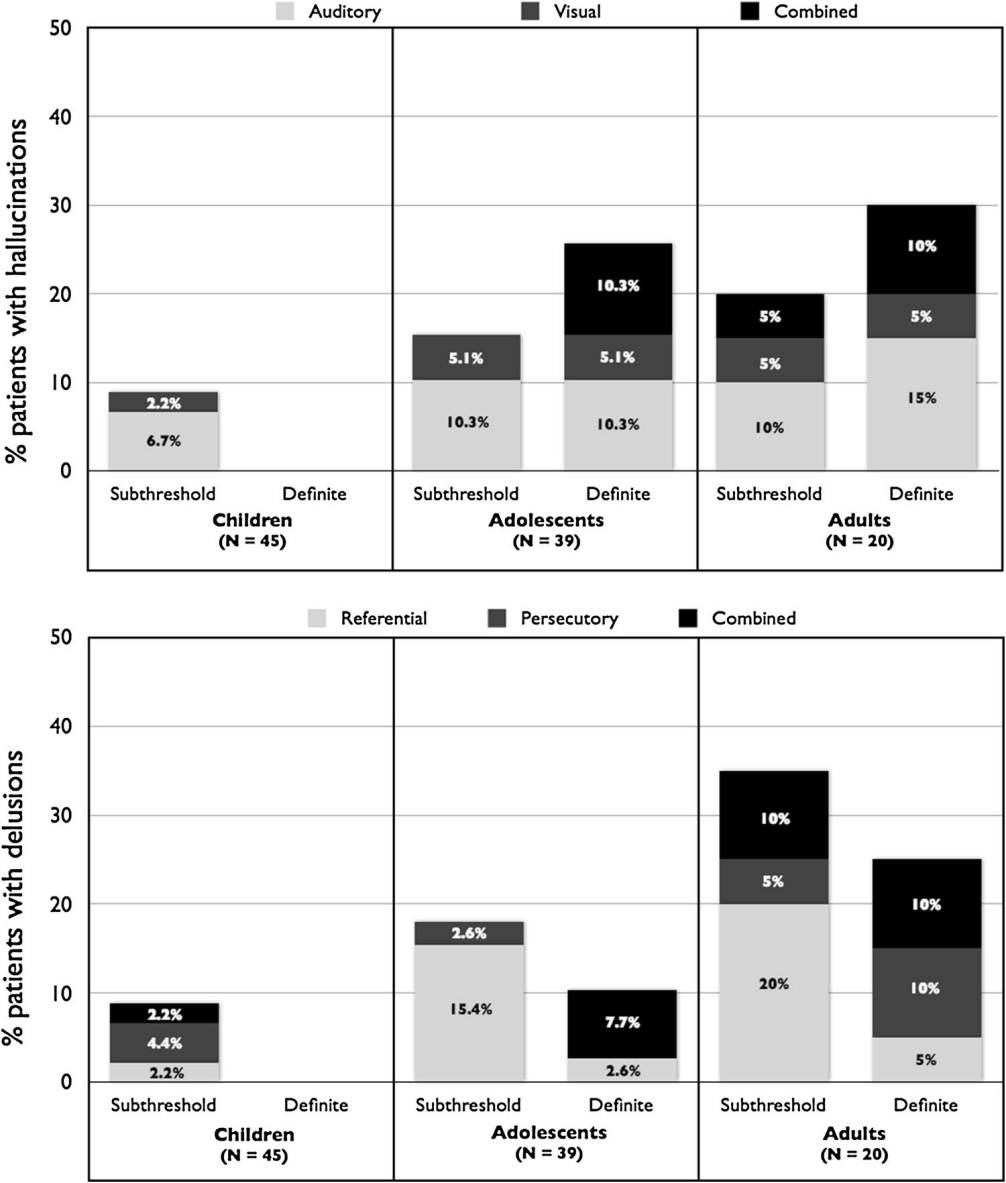
Frequency and characterization of positive psychotic symptoms by age group in the original sample (*N* = 104, cross-sectional design). **a** Percentage and modality of expression of subthreshold and definite hallucinations by age group; **b** percentage and main thematic of subthreshold and definite delusions by age group

Subthreshold hallucinations were observed in children as young as 6 years old, whereas definite hallucinations were seen from adolescence only (the youngest participant with definite hallucinations was aged 12). Auditory hallucinations were the most frequent in all age groups, followed by visual hallucinations (see Fig. 1a). When other types of hallucinations (e.g. olfactory) were present, it was always in combination with auditory hallucinations. Combined forms of hallucinations (i.e. hallucinations in two or more modalities) were rare in patients with subthreshold hallucinations (1 case), but frequent in patients with definite hallucinations (30 % of cases).

As for hallucinations, subthreshold delusions were observed in few children aged 10 years or higher, whereas definite delusions were rarer and seen from adolescence only (the youngest participant with definite delusions was aged 15) (see Fig. 1b). Ideas of reference (i.e. subthreshold delusions with a referential theme) and subthreshold persecutory delusions were the most frequent forms of subthreshold delusions at all ages (6.73 and 3.85 % of the total sample, respectively). Delusions of reference (i.e. definite delusions with a referential theme) and definite persecutory delusions also were the most frequent forms of definite delusions at all ages (each affecting 1.92 % of the total sample). When other forms of subthreshold or definite delusions were present (e.g. grandiose theme), it was always in combination with a referential or a persecutory theme.

#### Psychotic symptoms across different stages of psychosis

**Fig. 2:**
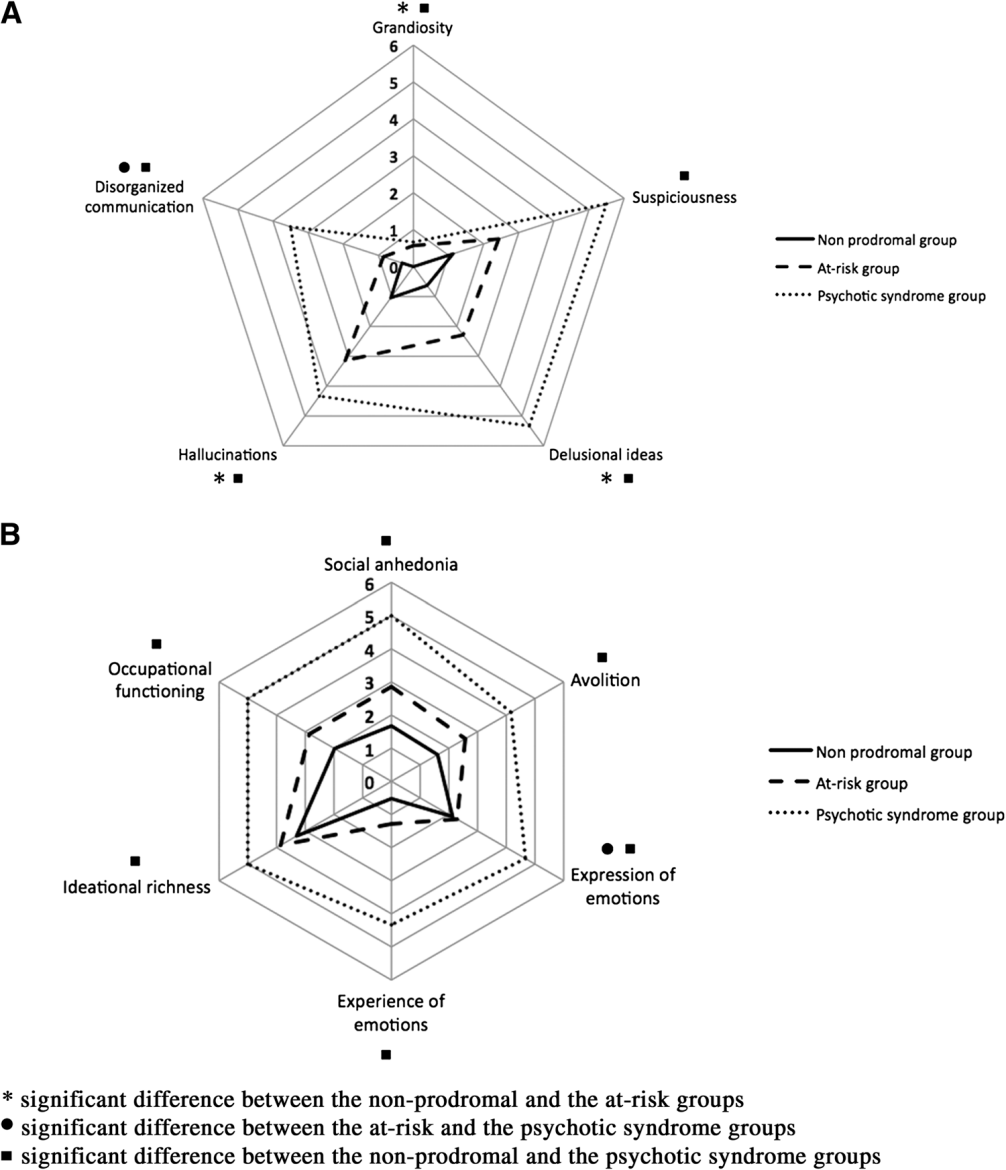
Mean severity of SIPS positive (**a**) and negative (**b**) symptoms according to clinical status. The mean score on each SIPS item for the three groups is provided at each corner of the graph. Significant differences are based on Kruskal–Wallis tests with pairwise comparisons of category

SIPS data were available in a subsample of 59 participants from the original cross-sectional sample of 104 individuals. According to the COPS 3.0. criteria, six participants (*m* = 21.55, SD = 6.99; 1 female) had a current psychotic syndrome and seven were in an at-risk mental state (*m* = 16.41, SD = 4.31; 2 females). The remaining 46 participants (*m* = 16.41, SD = 5.52; 30 females) comprised the non-prodromal group. Kruskal–Wallis omnibus tests on the SIPS positive and negative symptom scores were all significant (*p* < 0.05). Pairwise comparisons of category (with adjusted *p* values) revealed that individuals in an at-risk mental state had significantly higher scores on items P1 (delusional ideas) (rank difference = −17.590, *p* = 0.021), P3 (grandiosity) (rank difference = −8.286, *p* = 0.018) and P4 (hallucinations) (rank difference = −18.194, *p* = 0.016) compared to the non-prodromal group (see Fig. 2). They did not differ on items P2 (suspiciousness), P5 (disorganized communication) and on the six items assessing negative symptoms (all *p* > 0.05). The comparison between the at-risk and the psychotic syndrome groups revealed a significant difference in item P5 (disorganized communication) (rank difference = −21.500, *p* = 0.028) and item N3 (expression of emotions) (rank difference = −24.048, *p* = 0.029). Finally, individuals from the psychotic syndrome group had significantly higher scores on all positive and negative items than non-prodromal individuals (all *p* < 0.05).

### Longitudinal analyses

#### Developmental trajectory of positive psychotic symptoms

**Fig. 3:**
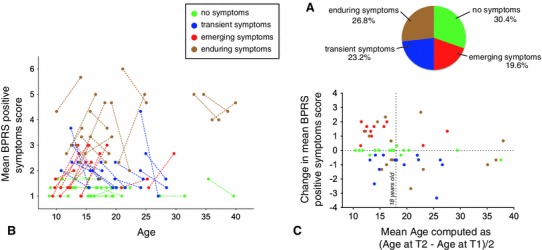
Longitudinal trajectories of the BPRS positive symptom score. **a** Percentage of the sample included in the four groups of patients: no positive psychotic symptoms, transient symptoms, emerging symptoms, and enduring symptoms. **b** Graphical representation of the BPRS positive symptom score at T1 and T2 evaluations in the four groups of patients. T1 and T2 scores of the same participant are connected with a *dashed line*. **c** Graphical representation of the BPRS positive symptom score change between the two visits. Significantly more patients with emerging symptoms were under the age of 18 years (9 patients under the age of 18 vs. 2 patients above the age of 18)

We characterized the different trajectories of positive psychotic symptoms using longitudinal data acquired on a subsample of 56 patients aged at least 12 years old at the second evaluation. A proportion of 30.4 % (*N* = 17) of patients showed no positive psychotic symptoms at either time point, 23.2 % (*N* = 13) displayed transient symptoms, 19.6 % (*N* = 11) showed emerging positive psychotic symptoms and 26.8 % (*N* = 15) displayed enduring positive psychotic symptoms (see Fig 3a). All the participants receiving antipsychotic medication either at T1 (*N* = 3) or T2 (*N* = 7) evaluations were part of the group of patients with enduring positive psychotic symptoms. For visualization purpose, the trajectories of the mean BPRS positive symptom score (mean severity score of BPRS hallucinatory behaviour, suspiciousness, and unusual thought content items) in the four groups are displayed in Fig. 3b. Changes in the mean BPRS positive symptom score between the two visits are also shown as a function of age (see Fig. 3c). A binomial test revealed that patients with emerging symptoms were significantly more often under the age of 18 years (9 patients under the age of 18 vs. 2 patients above the age of 18: one-tail *p* = 0.033). This was not the case for patients with no positive psychotic symptoms (one-tail *p* = 0.166), transient symptoms (one-tail *p* = 0.500), or enduring symptoms (one-tail *p* = 0.304).

#### Cognitive trajectories in patients with and without positive psychotic symptoms

**Fig. 4:**
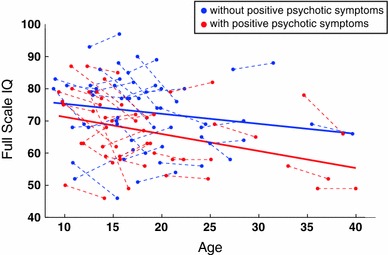
Developmental trajectory of full-scale IQ in participants with and without psychotic symptoms. The trajectories in the two groups have a significantly different intercept value

Finally, we compared the cognitive trajectories in individuals with (*N* = 26) and without (*N* = 30) positive psychotic symptoms at T2 (see Table 3). Contrary to our hypothesis, we observed no significant difference in the shape of the trajectories between the two groups. However, we observed a significant intercept difference for several cognitive domains. In most cases, the trajectories were linearly decreasing over time, with a significant intercept difference between the two groups in favour of individuals without positive psychotic symptoms (see Fig. 4). This included full-scale IQ (*p* = 0.034), the processing speed index (*p* = 0.027), and the delayed recognition memory index (*p* = 0.046). Other domains were stable with age, with a significant interecept difference in favour of patients without psychotic symptoms. This was the case for performance IQ (*p* = 0.011) and the perceptual organization index (*p* = 0.033).

**Table 3:** Mean (SD) cognitive scores at T1 and T2 in patients with and without positive psychotic symptoms at follow-up evaluation

Cognitive measures	Without positive psychotic symptoms at follow-up		With positive psychotic symptoms at follow-up	
	T1	T2	T1	T2
WISC-III/WAIS-III indices				
Full-Scale IQ	73.60 (11.54)	71.80 (11.39)	68.54 (11.59)	64.35 (9.98)
Verbal IQ	78.00 (14.28)	74.70 (14.06)	74.88 (14.10)	68.96 (13.07)
Performance IQ	73.73 (10.54)	72.97 (10.29)	67.85 (12.16)	65.12 (10.94)
Verbal comprehension index	82.10 (14.01)	78.80 (14.70)	77.77 (14.60)	72.85 (12.25)
Perceptual organization index	74.50 (10.66)	73.10 (10.56)	69.08 (11.82)	67.08 (11.11)
Processing Speed Index	82.43 (14.32)	80.30 (16.78)	75.12 (17.99)	70.31 (16.55)
CMS/WMS-III indices				
Visual immediate	81.13 (13.78)	79.20 (13.31)	74.36 (15.42)	74.04 (19.19)
Visual delayed	82.40 (16.84)	82.57 (11.26)	77.28 (15.51)	74.42 (18.26)
Auditory immediate	94.27 (15.09)	87.60 (14.94)	83.24 (17.52)	82.00 (17.09)
Auditory delayed	94.47 (16.08)	88.83 (15.11)	83.52 (19.37)	84.04 (17.74)
Auditory recognition delayed	97.17 (11.63)	89.80 (12.11)	89.40 (16.88)	85.57 (19.00)

The trajectories in the remaining cognitive domains did not significantly differ between the two groups. Some domains were stable with age (auditory immediate memory index, auditory delayed memory index, visual immediate memory index, and visual delayed memory index), whereas other declined with age (verbal IQ and verbal comprehension index).

## Discussion

In the present article, we characterized positive psychotic symptoms and schizophrenia spectrum disorders in a large cross-sectional sample of patients with 22q11DS. In accordance with our first hypothesis, we observed a high prevalence of positive psychotic symptoms and schizophrenia spectrum disorders in adolescents and adults with 22q11DS. In accordance with our second hypothesis and based on analyses performed using the SIPS, we observed that the severity of hallucinations distinguished between non-prodromal patients and at-risk patients. Our results also suggest that the severity of grandiosity and non-persecutory delusional ideas is also discriminant. However, the severity of negative symptoms did not differ between these two groups of patients. Using longitudinal data available in a subsample of 56 patients, we observed that 27 % of patients experienced positive psychotic symptoms of increasing severity over time and that these patients were aged below 18 years in the majority of cases. On the contrary, 20 % of the sample was characterized by a transient symptomatology. Finally, we identified several cognitive scores, such as full-scale IQ, processing speed, and verbal memory, as putative trait markers for the presence of psychotic symptoms. Contrary to our hypothesis, no state markers for the presence of psychotic symptoms were identified.

### Characterization of schizophrenia spectrum disorders and positive psychotic symptoms in 22q11DS

In the present sample, we observed a high prevalence of schizophrenia spectrum disorders in adults with 22q11DS, similar to what was typically observed in other research groups [e.g. 1]. Furthermore, we observed that the rate of schizophrenia spectrum disorders was also increased in the adolescent group (8 %), suggesting that early-onset psychosis in frequent in 22q11DS and may represent a clinical characteristic of the syndrome [31, 32]. Indeed, the onset occurred before the age of 18 years in 30 % of individuals with a schizophrenia spectrum disorder. Although the exact prevalence of early-onset schizophrenia (i.e. onset before 18 years) in schizophrenic patients without 22q11DS remains unknown, the 30 % prevalence observed in the present study is much higher than the estimated rate of early-onset schizophrenia [e.g. 33]. For this reason, child and adolescent psychiatrists unfamiliar with 22q11DS may misdiagnose psychotic symptoms in these adolescents. Because the duration of untreated psychosis is known to affect outcome [34], it thus becomes increasingly important to heighten practitioners’ awareness about the specificities of schizophrenia spectrum disorders in the context of 22q11DS.

When psychotic symptoms were examined in the same sample of patients, it appeared that a substantial proportion of them reported psychotic manifestations in the absence of a full-blown psychotic disorder and even at a very young age [see also 3, 31]. Taken together, subthreshold and definite positive psychotic symptoms were experienced by more than 50 % of adolescents and 75 % of adults with 22q11DS. When subthreshold and definite positive psychotic symptoms were examined separately, this revealed that subthreshold manifestations already emerged in few children, whereas definite symptoms were present from adolescence only. In general, hallucinations tended to emerge earlier than delusional ideas (the youngest patient experiencing definite hallucinations was aged 12 years, whereas the youngest patient experiencing definite delusions was aged 15). This goes in favour of Garety’s model of positive symptoms [35], which argues that delusional ideas account for the presence of anomalous experiences and therefore follow the onset of hallucinations in the majority of cases. The description of the phenomenology of positive psychotic symptoms in this population is generally consistent with the previous description of Vorstman et al. [32]. We observed that hallucinations were more frequently auditory than visual and that delusions were predominantly referential. Our results also suggest that combined forms of hallucinations and delusions were almost exclusively present in patients with definite psychotic symptoms and rarely observed in patients with subthreshold manifestations. Altogether, these data suggest that psychosis in 22q11DS should not be considered as an all-or-nothing phenomenon, but rather as a continuum of psychotic experiences of increasing severity. This also underlines the importance of carefully assessing psychotic manifestations in 22q11DS individuals, even in the paediatric population. However, our clinical experience suggests that these patients are often reluctant to share their experiences for fear of being judged negatively, particularly with their families. For this reason, clinicians should always spend time alone with the patient and investigate the presence of symptoms using detailed and oriented questions. In particular, a careful assessment of auditory hallucinations and ideas/delusions of reference should be performed.

### Evolution of the severity of psychotic symptoms over time

The comparison of positive and negative symptoms in participants at different stages of psychosis revealed that the severity of hallucinations, non-persecutory delusional ideas, and grandiosity discriminate between at-risk patients and non-prodromal individuals. Disorganized communication distinguished at-risk patients and individuals with a psychotic syndrome. These results suggest that hallucinations, delusional ideas such as mind reading, ideas of reference, or magical thinking are among the first positive symptoms that are likely to appear in 22q11DS youth. On the contrary, it suggests that more disorganized aspects of the behaviour are more likely to appear during the transition to a full-blown psychotic state. Based on our clinical experience with this population, we recommend instituting an antipsychotic medication to avoid an evolution of symptomatology towards disorganization when psychotic symptoms are present more than once a month or are described as intrusive. By contrast, we observed that the severity of negative symptoms did not discriminate at-risk patients from non-prodromal individuals. There are at least two possible interpretations for this result. On one hand, negative-like symptoms may be a characteristic of the 22q11DS clinical profile and may not be specifically related to the development of psychosis in this population. For example, these manifestations could be related to cognitive deficits. On the other hand, negative symptoms may predict the development of psychosis, while preceding the onset of positive symptoms. This interpretation is commensurate with Dominguez et al. [36], who suggest that negative symptoms appear several years before the onset of positive symptoms and predict their occurrence. Furthermore, negative symptoms have been shown to predict transition to schizophrenia [37, 38]. Future longitudinal studies in 22q11DS examining the predictive value of negative symptoms in the conversion to schizophrenia will help clarify these alternative interpretations.

In accordance with our third hypothesis, we observed that positive psychotic symptoms had different trajectories of evolution over time. Indeed, approximately one-third of the sample experienced emerging symptoms between T1 and T2 evaluations. These patients were below the age of 18 years in 82 % of cases, suggesting that the period of maximum risk for conversion to psychosis is relatively limited in time and occurs during adolescence in the great majority of cases. This should motivate clinicians to perform clinical evaluations at more regular intervals during this period and be particularly vigilant about symptom exacerbation. On the contrary, 20 % of the sample experienced transient psychotic symptoms during the 4-year interval, an interesting finding that has never been reported thus far in this population. As no patient from this subgroup received antipsychotic medication at T1 or T2 evaluations, one cannot assume that symptoms decreased due to a pharmacological intervention. It is either possible that the course of positive psychotic symptoms is naturally transient in some individuals with 22q11DS, or that symptoms were reduced during this period due to an active management of known risk factors for the development of psychotic symptoms (e.g. anxiety management, psychoeducation focused on cannabis use, etc.). Future studies on 22q11DS should further investigate the characteristics of this subgroup of patients, which could give important insights into adequate intervention strategies for the management of psychotic symptoms in this population.

### Associations between positive psychotic symptoms and cognitive functioning

We observed that most cognitive domains were linearly decreasing over time both in patients with and without positive psychotic symptoms. In accordance with other studies [e.g. 39], this suggests that 22q11DS is associated with a general cognitive decline over time, which particularly affects verbal reasoning abilities and processing speed. However, perceptual organization, verbal and visual memory appear to be more stable cognitive domains in this population, given that their trajectory remained constant with age in all participants.

Contrary to our hypothesis, we were not able to identify putative state markers for the onset of psychotic symptoms in this population. Indeed, we did not observe significant difference in the shape of the cognitive trajectories between patients with and without psychotic symptoms. However, and as hypothesized, we observed that several cognitive domains were significantly impaired in patients with psychotic symptoms, but that their developmental trajectory was identical to the one observed in patients without psychotic symptoms (referred to as intercept difference). This suggests that low scores in these domains from childhood are associated with an increased risk to develop psychotic symptoms later in life and may represent early trait markers for psychotic symptoms. In particular, this was the case for global reasoning abilities (full-scale IQ), processing speed, and perceptual organization. Some of these domains have already been reported as risk factors for psychotic symptoms in previous 22q11DS longitudinal studies [13, 14]. It may be the case that children with low cognitive abilities in the context of the 22q11DS [i.e. patients scoring in the range of intellectual disability (IQ <70) vs. patients scoring in the borderline range of functioning or higher (IQ >70)] have more difficulties coping with their environment and experience it as being more unpredictable [40]. This, in turn, could predispose to the development of anxiety, which is a known important risk factor for schizophrenia [4]. However, this interpretation remains speculative and should be tested in future studies.

## Conclusions

Few important limitations should be considered when interpreting our results. First, although our longitudinal cohort is one of the largest in Europe, the sample size is still insufficient to perform a detailed assessment of participants with a full-blown schizophrenia spectrum disorder (vs. participants with psychotic symptoms). Further, there are still limited opportunities for pooling data among 22q11DS samples, partly due to the use of different assessment tools. Several groups are currently working on a 22q11DS Consortium to bring cohorts together and find solutions to overcome this issue [41]. Another limitation relates to the sole use of the SIPS criteria to assess risk for psychosis. Further studies on 22q11DS should also include other at-risk criteria, such as the presence of basic symptoms or anomalies of self-experience. Finally, the present study included some adult participants with 22q11DS who participated in our research following psychiatric difficulties. This may have increased the prevalence of psychiatric disorders in this subgroup. Future studies should only include adults who were first followed longitudinally as adolescents to avoid this ascertainment bias.

Based on the results of the present study and our clinical experience, several final recommendations can be made. The presence of psychotic symptoms should be explicitly investigated and attentively monitored in 22q11DS individuals to reduce the duration of untreated psychosis and improve outcome. Risk for schizophrenia should be evaluated in light of existing clinical and cognitive risk factors (e.g. low cognitive abilities, presence of an anxiety disorder). Clinicians should be aware that the exacerbation of positive psychotic symptoms occurs in most cases during adolescence.

## References

[1] Murphy K, Jones L, Owen M (1999). High rates of schizophrenia in adults with velo-cardio-facial syndrome. Arch Gen Psychiatry.

[2] WHO (1996) Schizophrenia and public health. Nations for mental health

[3] Debbané M, Glaser B, David MK, Feinstein C, Eliez S (2006). Psychotic symptoms in children and adolescents with 22q11.2 deletion syndrome: neuropsychological and behavioral implications. Schizophr Res.

[4] Gothelf D, Feinstein C, Thompson T, Gu E, Penniman L, Van Stone E, Kwon H, Eliez S, Reiss AL (2007). Risk factors for the emergence of psychotic disorders in adolescents with 22q11.2 deletion syndrome. Am J Psych.

[5] Armando M, Girardi P, Vicari S, Menghini D, Digilio MC, Pontillo M, Saba R, Mazzone L, Lin A, Klier CM, Schäfer MR, Amminger GP (2012). Adolescents at ultra-high risk for psychosis with and without 22q11 deletion syndrome: a comparison of prodromal psychotic symptoms and general functioning. Schizophr Res.

[6] Schneider M, Van der Linden M, Glaser B, Rizzi E, Dahoun SP, Hinard C, Bartoloni L, Antonarakis SE, Debbané M, Eliez S (2012). Preliminary structure and predictive value of attenuated negative symptoms in 22q11.2 deletion syndrome. Psychiat Res.

[7] Lucas S, Fitzgerald D, Redoblado-Hodge MA, Anderson J, Sanbrook M, Harris A, Brennan J (2004). Neuropsychological correlates of symptom profiles in first episode schizophrenia. Schizophr Res.

[8] Rhinewine JP, Lencz T, Thaden EP, Cervellione KL, Burdick KE, Henderson I, Bhaskar S, Keehlisen L, Kane J, Kohn N, Fisch GS, Bilder RM, Kumra S (2005). Neurocognitive profile in adolescents with early-onset schizophrenia: clinical correlates. Biol Psychiatry.

[9] Becker HE, Nieman DH, Wiltink S, Dingemans PM, van de Fliert JR, Velthorst E, de Haan L, van Amelsvoort TA, Linszen DH (2010). Neurocognitive functioning before and after the first psychotic episode: does psychosis result in cognitive deterioration?. Psychol Med.

[10] Keefe RSE, Perkins DO, Gu H, Zipursky RB, Christensen BK, Lieberman JA (2006). A longitudinal study of neurocognitive function in individuals at-risk for psychosis. Schizophr Res.

[11] Lencz T, Smith CW, McLaughlin D, Auther A, Nakayama E, Hovey L, Cornblatt BA (2006). Generalized and specific neurocognitive deficits in prodromal schizophrenia. Biol Psychiatry.

[12] Frommann I, Pukrop R, Brinkmeyer J, Bechdolf A, Ruhrmann S, Berning J, Decker P, Riedel M, Möller H-J, Wölwer W, Gaebel W, Klosterkötter J, Maier W, Wagner M (2011). Neuropsychological profiles in different at-risk states of psychosis: executive control impairment in the early- and additional memory dysfunction in the late-prodromal state. Schizophr Bull.

[13] Antshel KM, Shprintzen R, Fremont W, Higgins AM, Faraone SV, Kates WR (2010). Cognitive and psychiatric predictors to psychosis in velocardiofacial syndrome: a 3-year follow-up study. J Am Acad Child Adolesc Psychiatry.

[14] Gothelf D, Eliez S, Thompson T, Hinard C, Penniman L, Feinstein C, Kwon H, Jin S, Jo B, Antonarakis SE, Morris MA, Reiss AL (2005). COMT genotype predicts longitudinal cognitive decline and psychosis in 22q11.2 deletion syndrome. Nat Neurosci.

[15] Green T, Gothelf D, Glaser B, Debbane M, Frisch A, Kotler M, Weizman A, Eliez S (2009). Psychiatric disorders and intellectual functioning throughout development in velocardiofacial (22q11.2 deletion) syndrome. J Am Acad Child Adolesc Psychiatry.

[16] Shapiro DI, Cubells JF, Ousley OY, Rockers K, Walker EF (2011). Prodromal symptoms in adolescents with 22q11.2 deletion syndrome and schizotypal personality disorder. Schizophr Res.

[17] Schaer M, Debbané M, Bach Cuadra M, Ottet M-C, Glaser B, Thiran J-P, Eliez S (2009). Deviant trajectories of cortical maturation in 22q11.2 deletion syndrome (22q11DS): a cross-sectional and longitudinal study. Schizophr Res.

[18] Reich W (2000). Diagnostic interview for children and adolescents (DICA). J Am Acad Child Adolesc Psychiatry.

[19] First M, Spitzer R, Gibbon M, Williams J (1996) Structured clinical interview for the DSM-IV-TR axis I disorders (SCID-I). Biometrics Research, New York State Psychiatric Institute, New York

[20] Kaufman J, Birmaher B, Brent D, Rao U (1997). Schedule for affective disorders and schizophrenia for school-age children-present lifetime version (K-SADS-PL): initial reliability and validity data. J Am Acad Child Adolesc Psychiatry.

[21] McGlashan TH, Miller TJ, Woods SW (2001) Structured Interview for Prodromal Syndromes (SIPS; Version 3.0, unpublished manuscript). PRIME Research Clinic, Yale University, School of Medicine, New Heaven, Connecticut

[22] Miller TJ, McGlashan TH, Rosen JL, Cadenhead K, Cannon T, Ventura J, McFarlane W, Perkins DO, Pearlson GD, Woods SW (2003). Prodromal assessment with the structured interview for prodromal syndromes and the scale of prodromal symptoms: predictive validity, interrater reliability, and training to reliability. Schizophr Bull.

[23] Overall J, Gorham D (1962). The brief psychiatric rating scale. Psychol Rep.

[24] Nicholson IR, Chapman JE, Neufeld RW (1995). Variability in BPRS definitions of positive and negative symptoms. Schizophr Res.

[25] Wechsler D (1991) Wechsler Intelligence Scale for Children, 3rd edn. Manual. The Psychological Corporation, San Antonio

[26] Cohen M (1997). Children’s memory scale.

[27] Wechsler D (1997) Wechsler Adult Intelligence Scale, 3rd edn. Administration and scoring manual. The Psychological Corporation, San Antonio

[28] Wechsler D (1987). Manual for the Wechsler Memory Scale—revised.

[29] Dedrick RF, Ferron JM, Hess MR, Hogarty KY, Kromrey JD, Lang TR, Niles JD, Lee RS (2009). Multilevel modeling: a review of methodological issues and applications. Rev Educ Res.

[30] Peng H, Lu Y (2012). Model selection in linear mixed effect models. J Multivariate Anal.

[31] Baker KD, Skuse DH (2005). Adolescents and young adults with 22q11 deletion syndrome: psychopathology in an at-risk group. Br J Psychiatry.

[32] Vorstman JAS, Morcus MEJ, Duijff SN, Klaassen PWJ, Heineman-de Boer JA, Beemer FA, Swaab H, Kahn RS, van Engeland H (2006) The 22q11.2 deletion in children: high rate of autistic disorders and early onset of psychotic symptoms. J Am Acad Child Adolesc Psychiatry 45 (9):1104–1113 10.1097/01.chi.0000228131.56956.c1 16926618

[33] Kumra S (2008). Digging deeper using neuroimaging tools reveals important clues to early-onset schizophrenia. J Am Acad Child Adolesc Psychiatry.

[34] Marshall M, Lewis S, Lockwood A, Drake R, Jones P, Croudace T (2005). Association between duration of untreated psychosis and outcome in cohorts of first-episode patients: a systematic review. Arch Gen Psychiatry.

[35] Garety PA, Kuipers E, Fowler D, Freeman D, Bebbington PE (2001). A cognitive model of the positive symptoms of psychosis. Psychol Med.

[36] Dominguez MG, Saka MC, can Saka M, Lieb R, Wittchen HU, van Os J (2010) Early expression of negative/disorganized symptoms predicting psychotic experiences and subsequent clinical psychosis: a 10-year study. Am J Psych 167 (9):1075–1082 10.1176/appi.ajp.2010.09060883 20634371

[37] Riecher-Rössler A, Pflueger MO, Aston J, Borgwardt SJ, Brewer WJ, Gschwandtner U, Stieglitz R-D (2009). Efficacy of using cognitive status in predicting psychosis: a 7-year follow-up. Biol Psychiatry.

[38] Velthorst E, Nieman DH, Becker HE, van de Fliert R, Dingemans PM, Klaassen R, de Haan L, van Amelsvoort T, Linszen DH (2009). Baseline differences in clinical symptomatology between ultra high risk subjects with and without a transition to psychosis. Schizophr Res.

[39] Duijff SN, Klaassen PWJ, de Veye HFNS, Beemer FA, Sinnema G, Vorstman JAS (2012). Cognitive development in children with 22q11.2 deletion syndrome. Br J Psychiatry.

[40] Beaton EA, Simon TJ (2011). How might stress contribute to increased risk for schizophrenia in children with chromosome 22q11.2 deletion syndrome?. J Neurodev Disord.

[41] Bassett AS, McDonald-McGinn DM, Devriendt K, Digilio MC, Goldenberg P, Habel A, Marino B, Oskarsdottir S, Philip N, Sullivan K, Swillen A, Vorstman J (2011). Practical guidelines for managing patients with 22q11.2 deletion syndrome. J Pediatr.

